# Supplementary material for: Novel Antibody–Peptide Binding Assay Indicates Presence of Immunoglobulins against EGFR Phospho-Site S1166 in High-Grade Glioma
Source: Int J Mol Sci. 2022 May 2;23(9):5061. doi: 10.3390/ijms23095061 (PMC9100080; doi:10.3390/ijms23095061)

Supplemental Figure S1: LC-MS Peak plots of DNP feasibility experiment

LC-MS chromatograms (PRM) of DNP-labelled (Figures S1A and S2B) and unlabelled (Figures S1C and S1D) peptides from Ab-peptide binding experiment with anti-DNP present (S1A and S1C) or absent (S1B and S1D). Each peptide was measured in 4 fractions: IB = IgG-bound, UB = unbound, FB1 = filter bound 1 fractions, and FB2 = filter bound 2 fraction. Colors in the chromatogram plots indicate the various fragments detected and used for quantification.

Figure S1A: DNP-labelled peptides and anti-DNP present

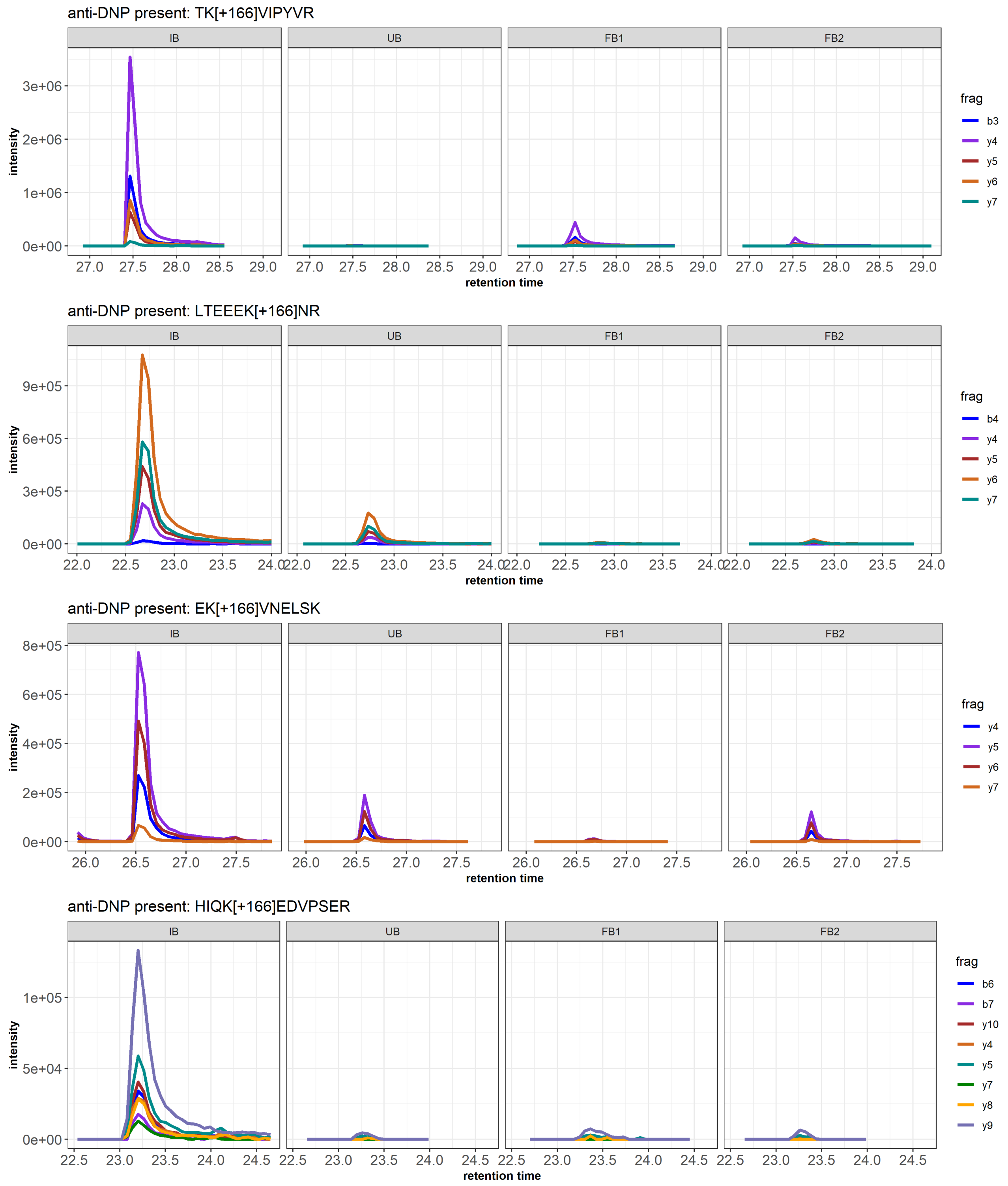

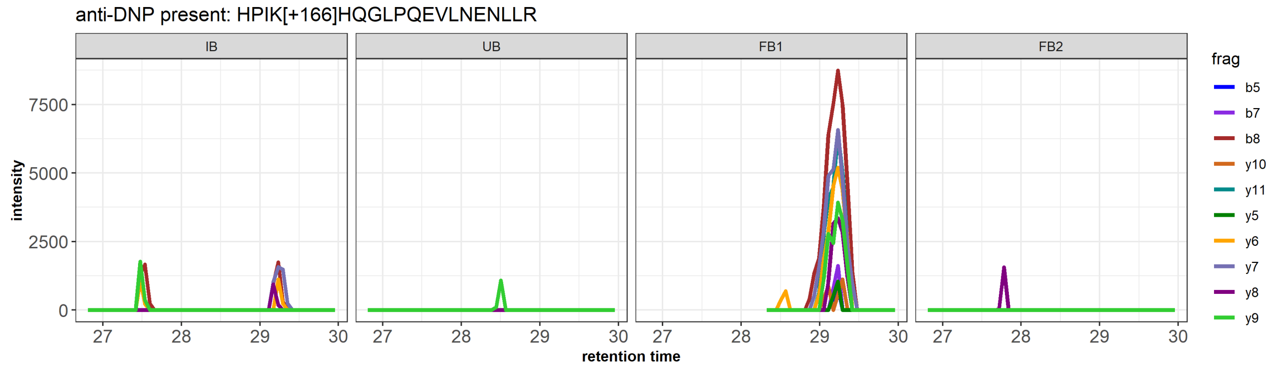
anti-DNP present: HPIK[+166]HQGLPQEVLNENLLR
IB
UB
FB1
FB2
intensity
0
2500
5000
7500
27
28
29
30
retention time
frag
b5
b7
b8
y10
y11
y5
y6
y7
y8
y9

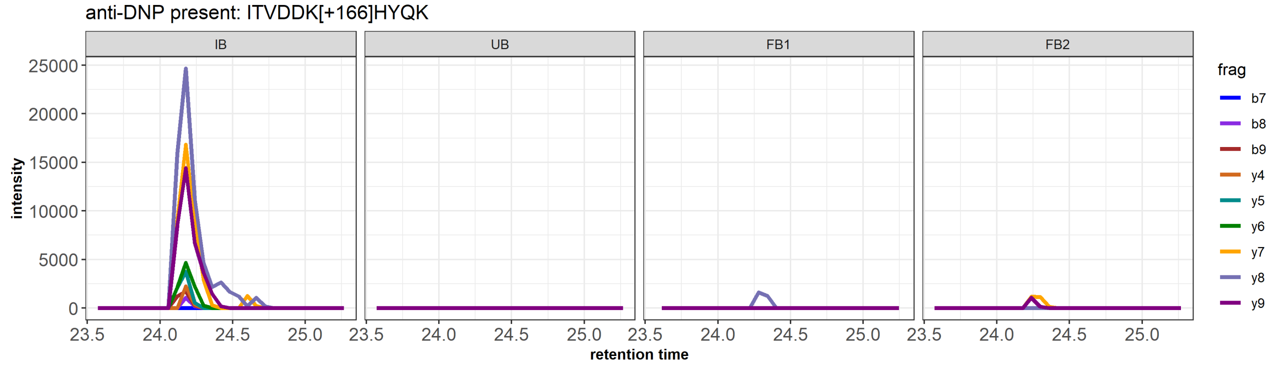
anti-DNP present: ITVDDK[+166]HYQK
IB
UB
FB1
FB2
intensity
0
5000
10000
15000
20000
25000
23.5
24.0
24.5
25.0
retention time
frag
b7
b8
b9
y4
y5
y6
y7
y8
y9

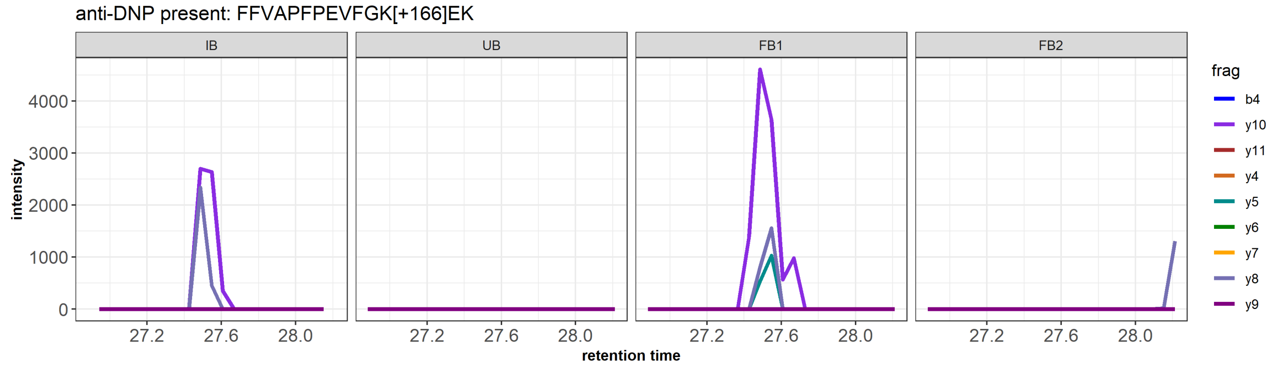
anti-DNP present: FFVAPFPEVFGK[+166]EK
IB
UB
FB1
FB2
intensity
0
1000
2000
3000
4000
27.2
27.6
28.0
retention time
frag
b4
y10
y11
y4
y5
y6
y7
y8
y9

Figure S1B: unlabelled peptides and anti-DNP present

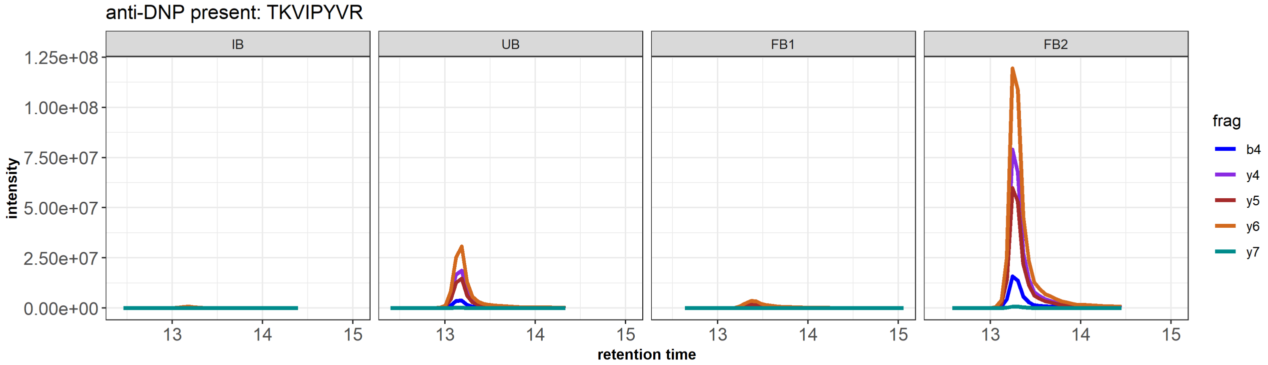

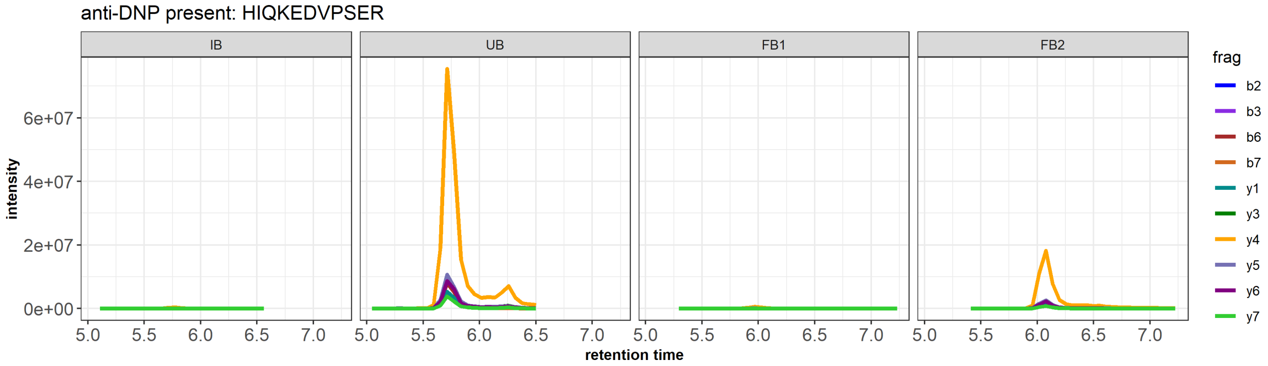

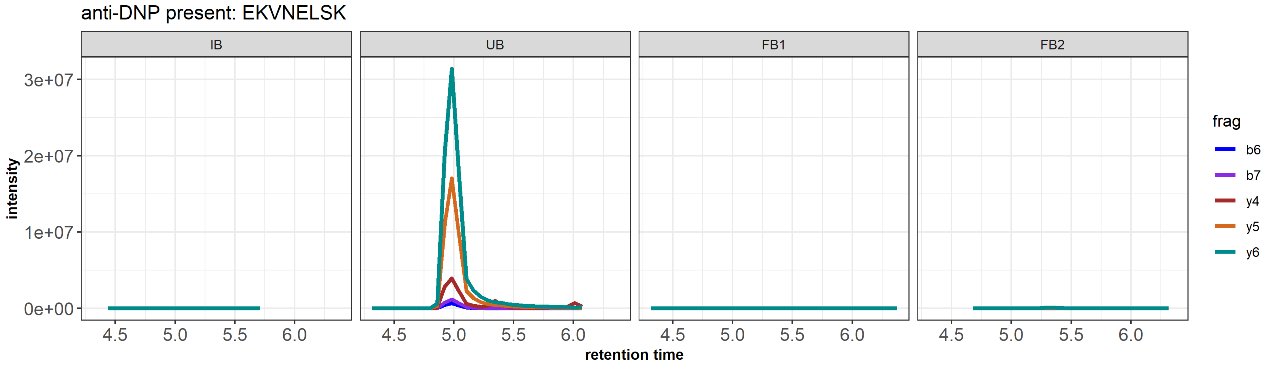

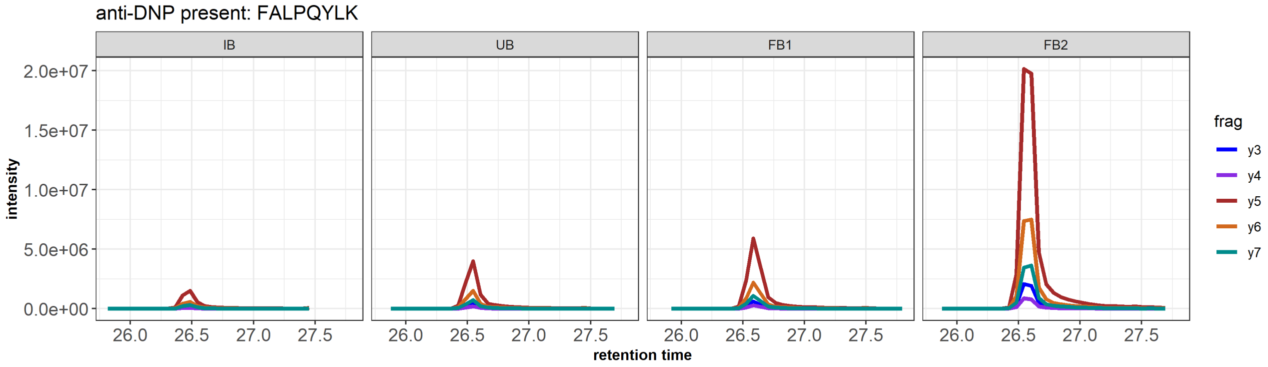

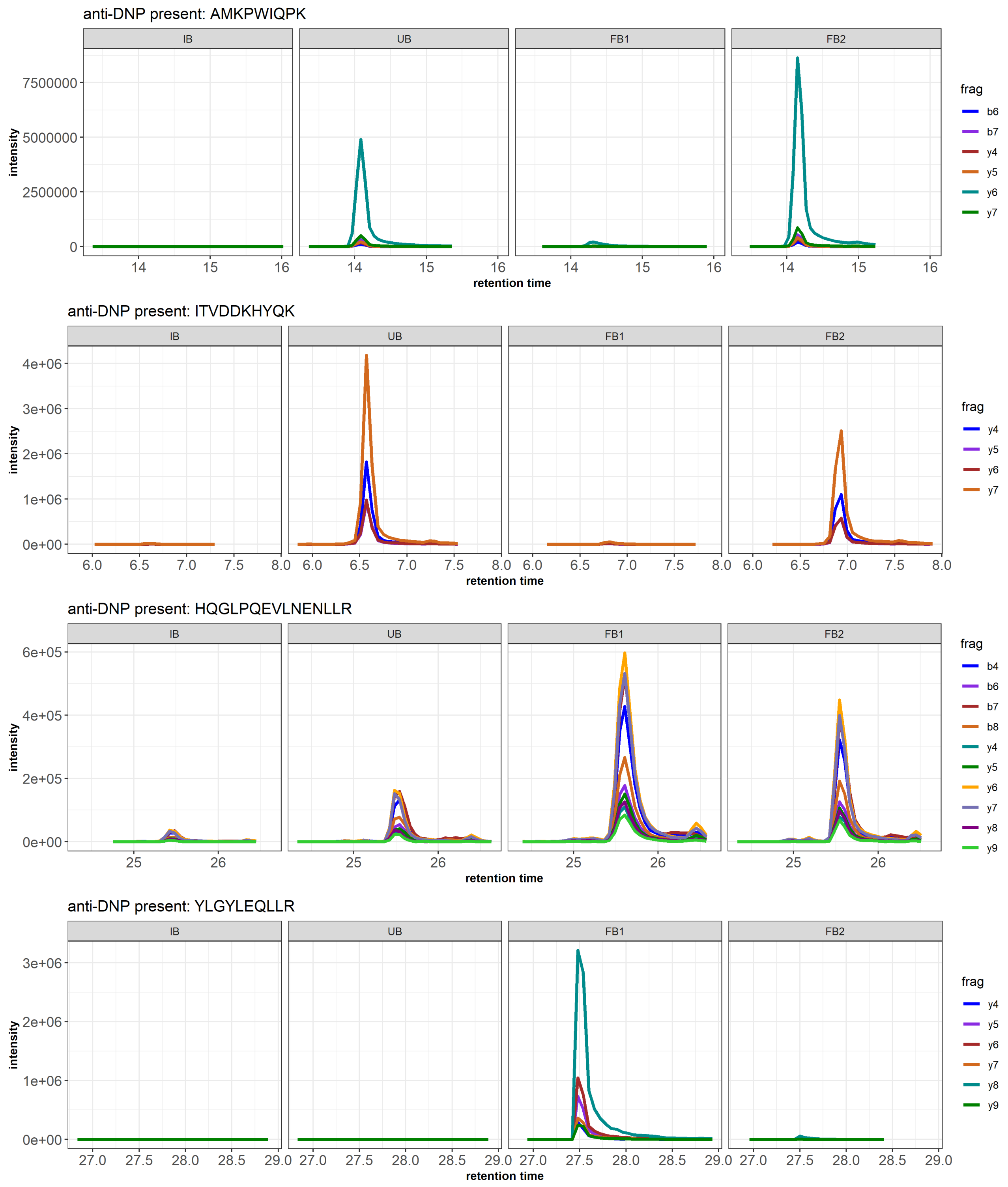

anti-DNP present: AMKPWIQPK
IB
UB
FB1
FB2
intensity
7500000
5000000
2500000
0
14
15
16
retention time
frag
b6
b7
y4
y5
y6
y7
anti-DNP present: ITVDDKHYQK
4e+06
3e+06
2e+06
1e+06
0e+00
6.0
6.5
7.0
7.5
8.0
frag
y4
y5
y6
y7
anti-DNP present: HQGLPQEVLNENLLR
6e+05
4e+05
2e+05
0e+00
25
26
frag
b4
b6
b7
b8
y4
y5
y6
y7
y8
y9
anti-DNP present: YLGYLEQLLR
3e+06
2e+06
1e+06
0e+00
27.0
27.5
28.0
28.5
29.0
frag
y4
y5
y6
y7
y8
y9

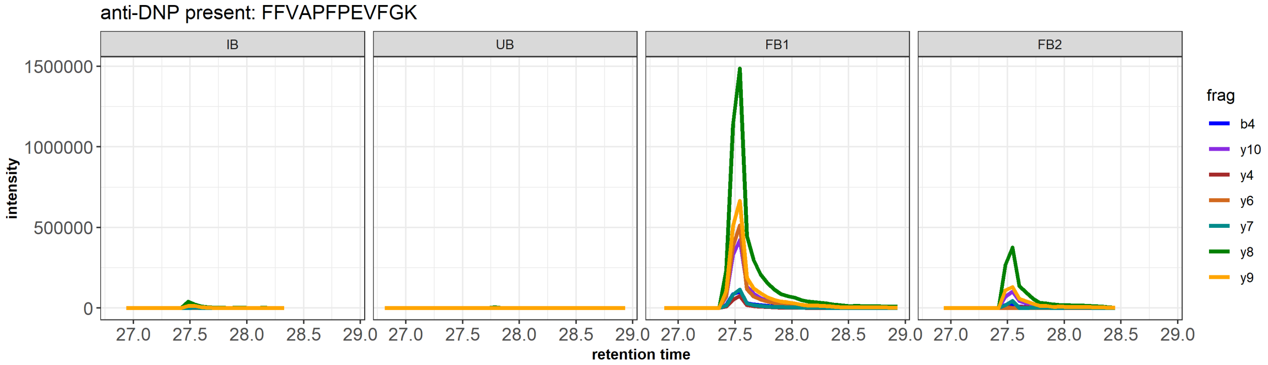
anti-DNP present: FFVAPFPEVFGK
IB
UB
FB1
FB2
intensity
1500000
1000000
500000
0
27.0
27.5
28.0
28.5
29.0
retention time
frag
b4
y10
y4
y6
y7
y8
y9

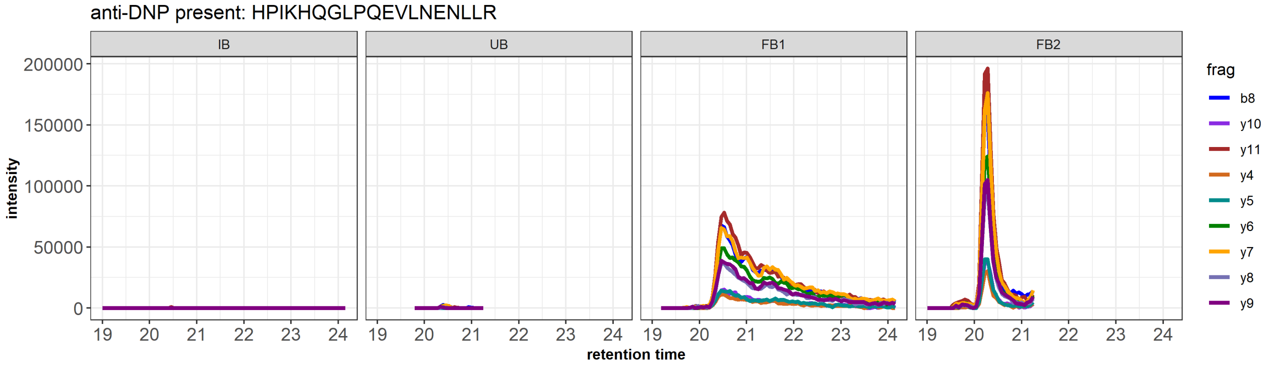
anti-DNP present: HPIKHQGLPQEVLNENLLR
IB
UB
FB1
FB2
intensity
200000
150000
100000
50000
0
19
20
21
22
23
24
retention time
frag
b8
y10
y11
y4
y5
y6
y7
y8
y9

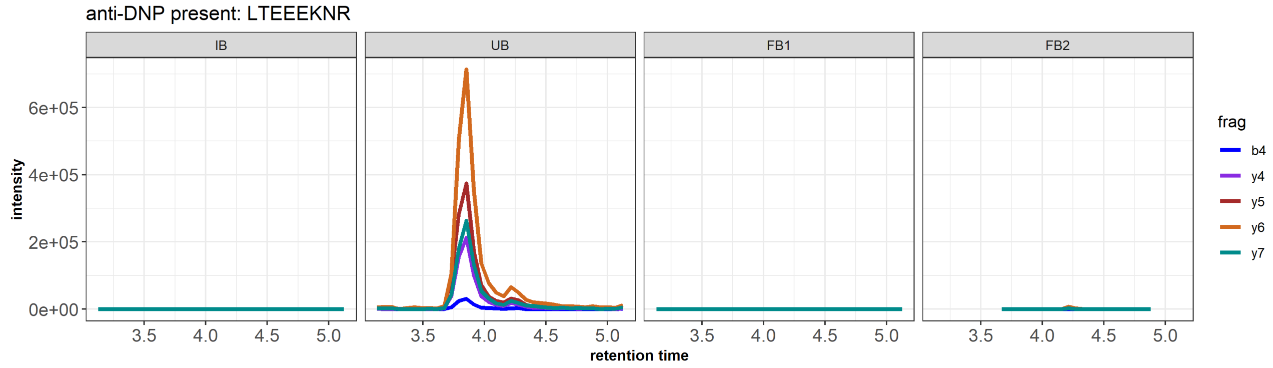
anti-DNP present: LTEEEKNR
IB
UB
FB1
FB2
intensity
6e+05
4e+05
2e+05
0e+00
3.5
4.0
4.5
5.0
retention time
frag
b4
y4
y5
y6
y7

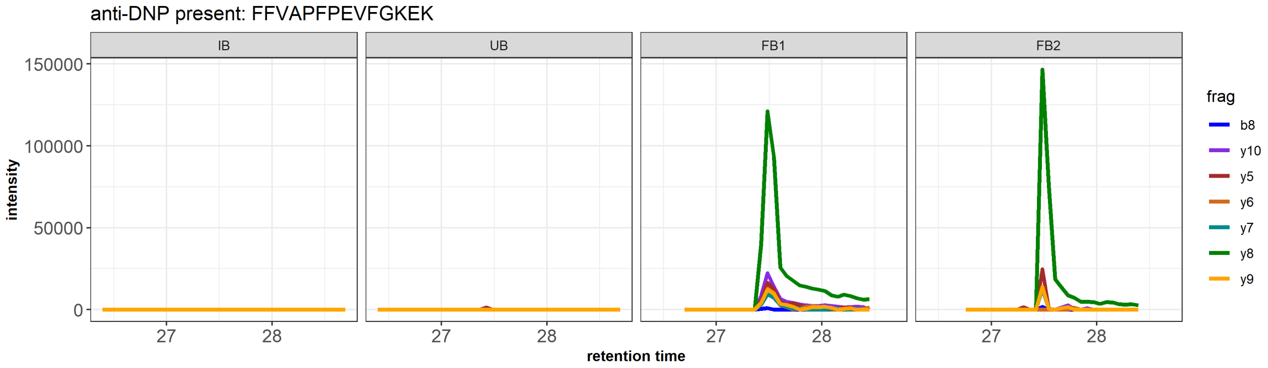
anti-DNP present: FFVAPFPEVFGKEK
IB
UB
FB1
FB2
intensity
150000
100000
50000
0
27
28
retention time
frag
b8
y10
y5
y6
y7
y8
y9

Figure S1C: DNP-labelled peptides and anti-DNP absent

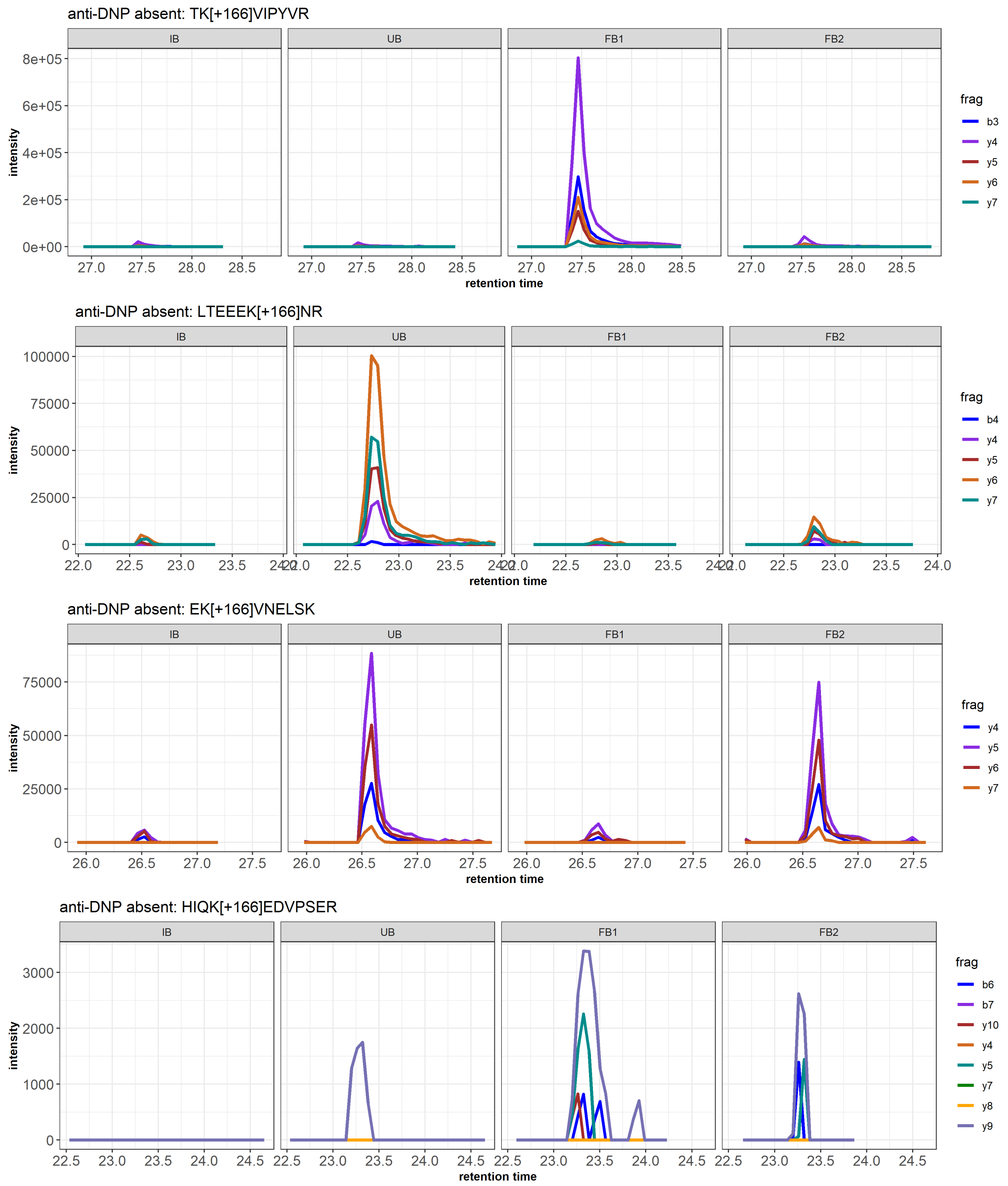

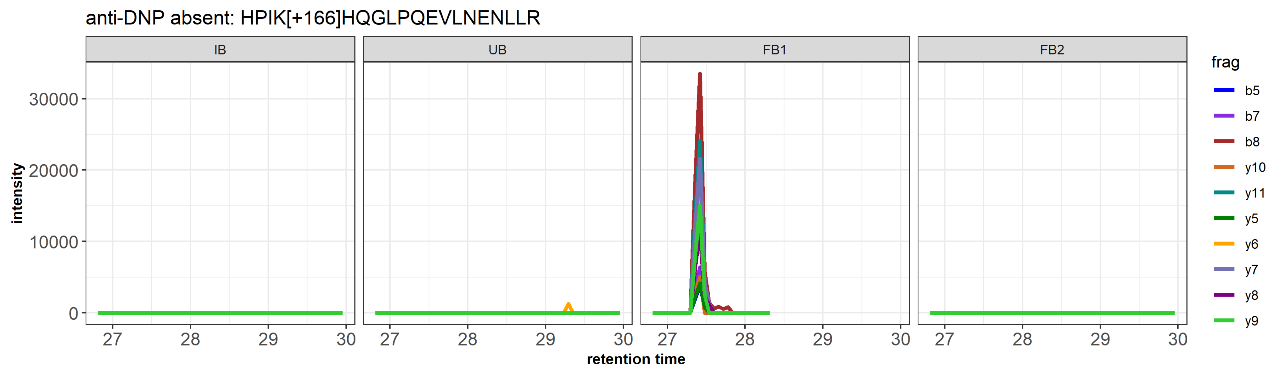
anti-DNP absent: HPIK[+166]HQGLPQEVLNENLLR
IB
UB
FB1
FB2
intensity
retention time
frag
b5
b7
b8
y10
y11
y5
y6
y7
y8
y9

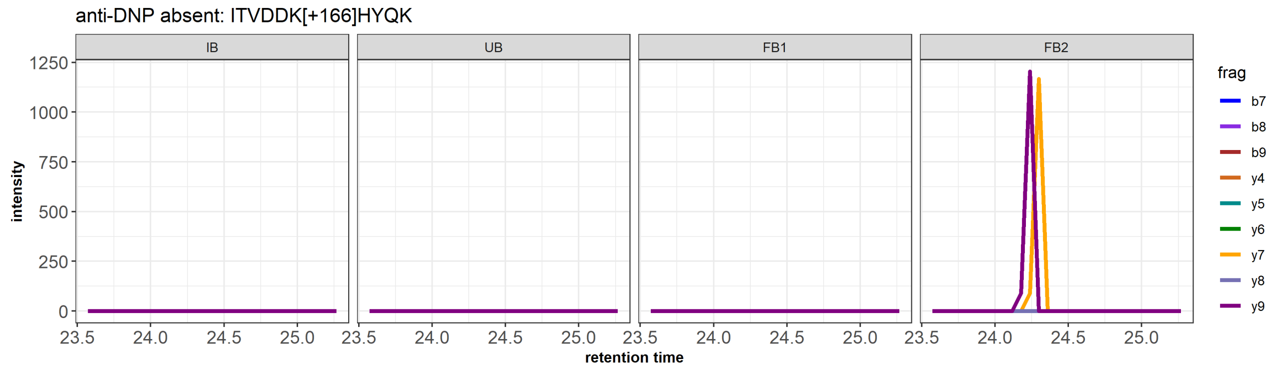
anti-DNP absent: ITVDDK[+166]HYQK
IB
UB
FB1
FB2
intensity
retention time
frag
b7
b8
b9
y4
y5
y6
y7
y8
y9

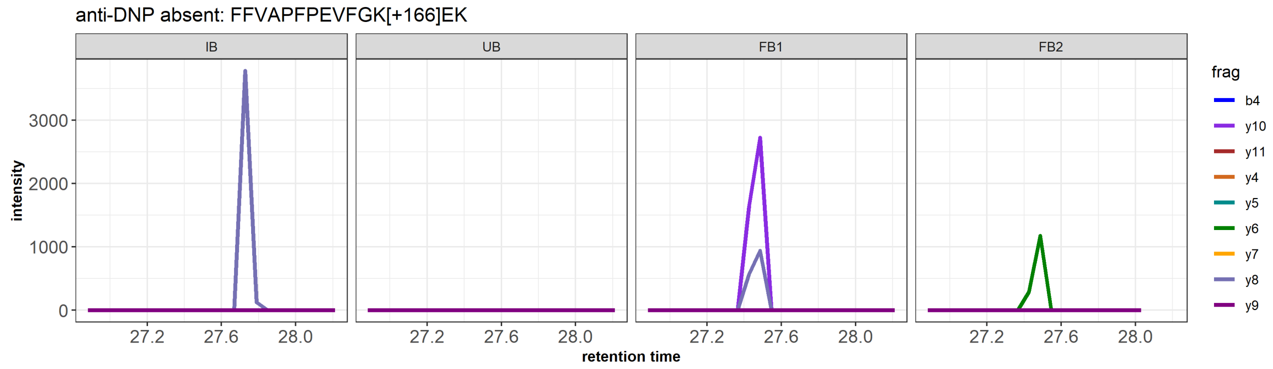
anti-DNP absent: FFVAPFPEVFGK[+166]EK
IB
UB
FB1
FB2
intensity
retention time
frag
b4
y10
y11
y4
y5
y6
y7
y8
y9

Figure S1D: unlabelled peptides and anti-DNP absent

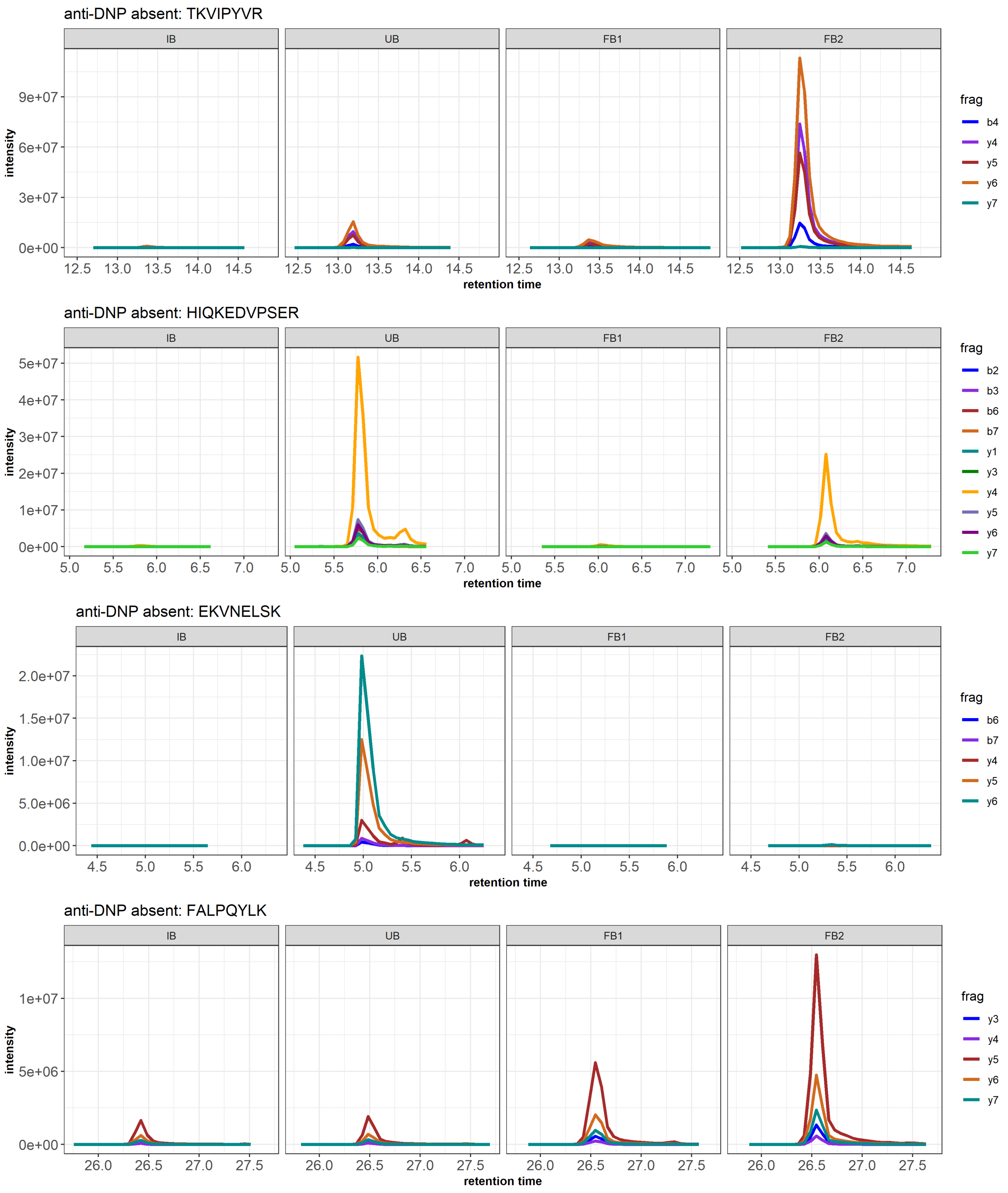

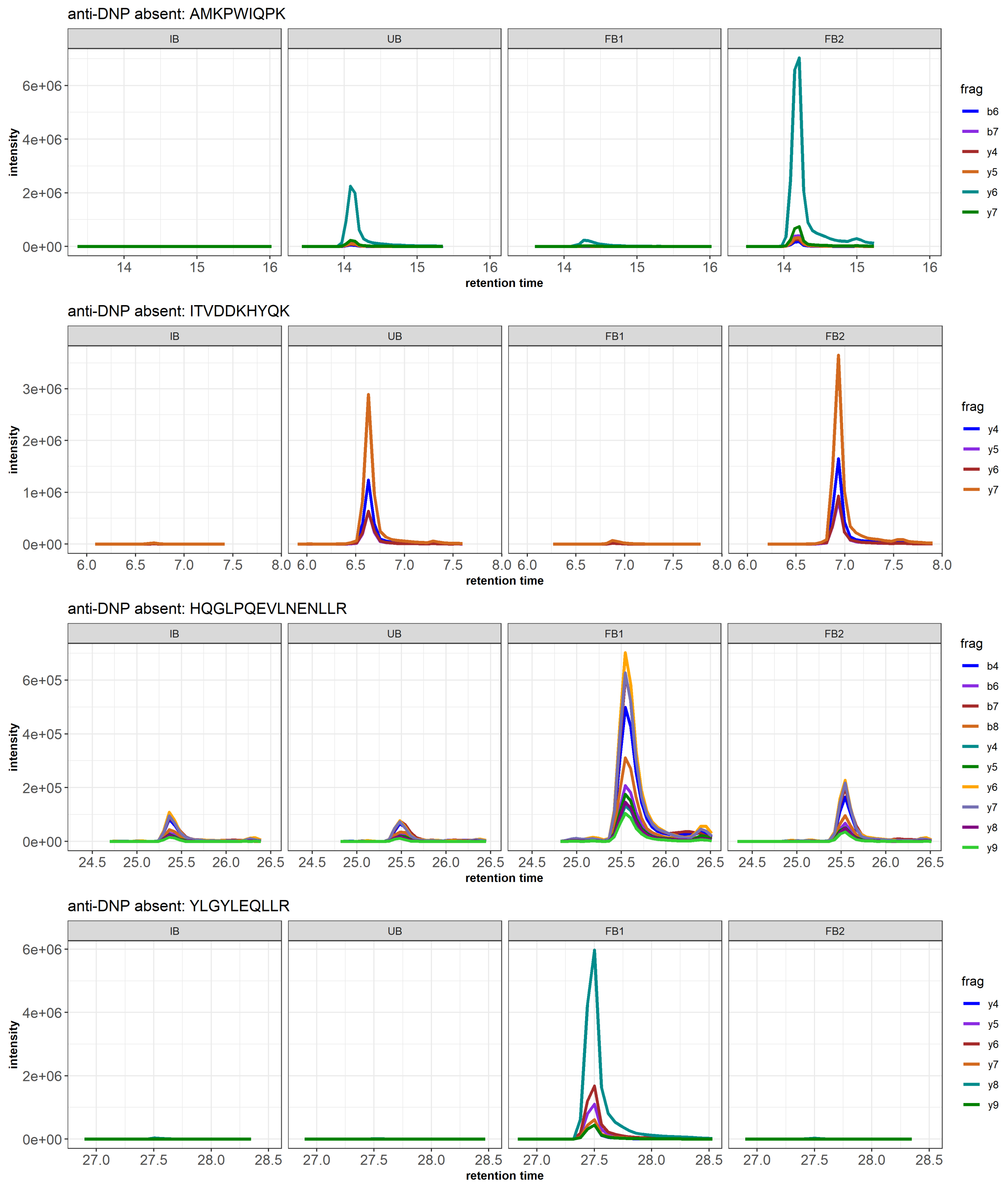
anti-DNP absent: AMKPWIQPK
IB
UB
FB1
FB2
intensity
6e+06
4e+06
2e+06
0e+00
14
15
16
retention time
frag
b6
b7
y4
y5
y6
y7
anti-DNP absent: ITVDDKHYQK
3e+06
2e+06
1e+06
0e+00
6.0
6.5
7.0
7.5
8.0
y4
y5
y6
y7
anti-DNP absent: HQGLPQEVLNENLLR
6e+05
4e+05
2e+05
0e+00
24.5
25.0
25.5
26.0
26.5
b4
b6
b7
b8
y4
y5
y6
y7
y8
y9
anti-DNP absent: YLGYLEQLLR
27.0
27.5
28.0
28.5
y4
y5
y6
y7
y8
y9

anti-DNP absent: FFVAPFPEVFGK

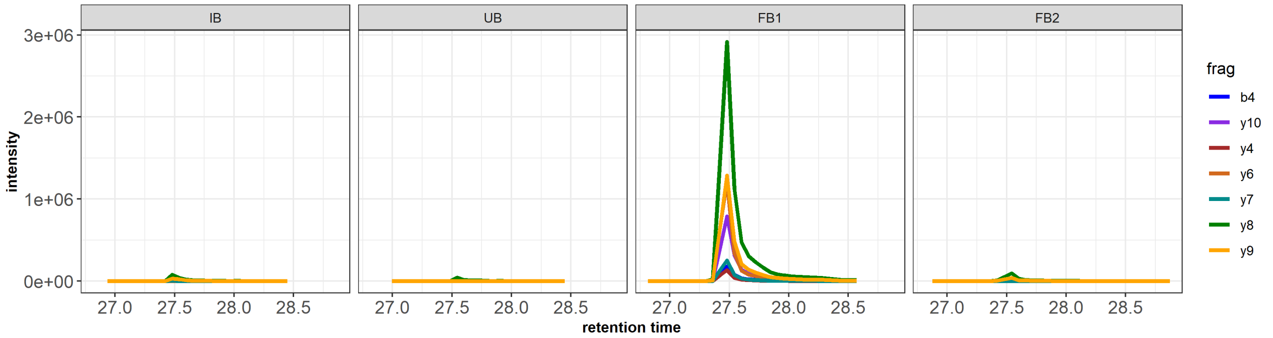

anti-DNP absent: HPIKHQGLPQEVLNENLLR

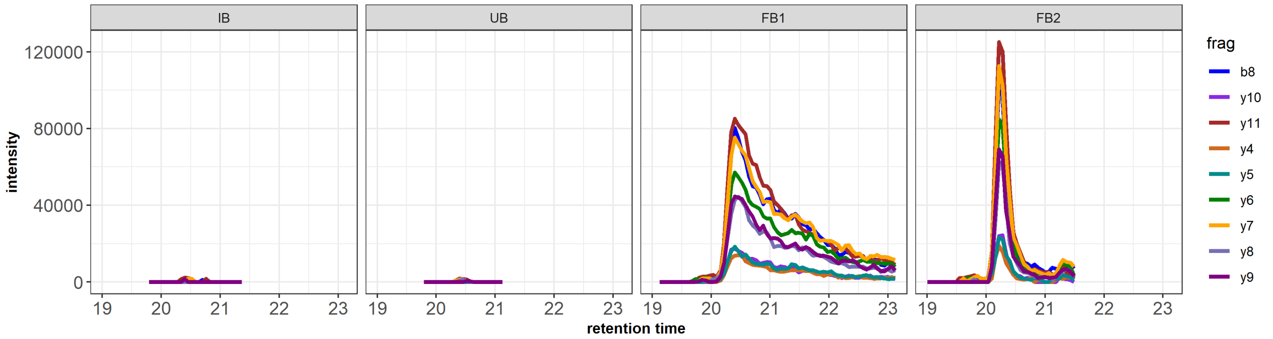

anti-DNP absent: LTEEEKNR

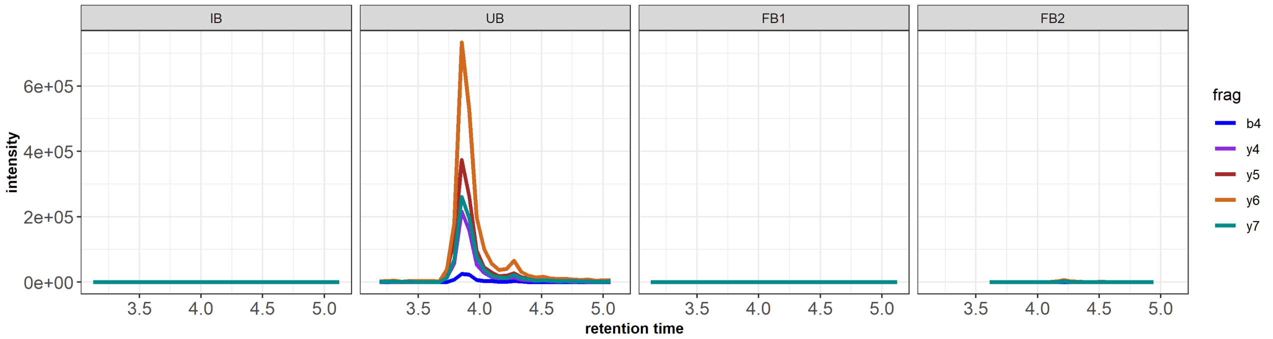

anti-DNP absent: FFVAPFPEVFGKEK

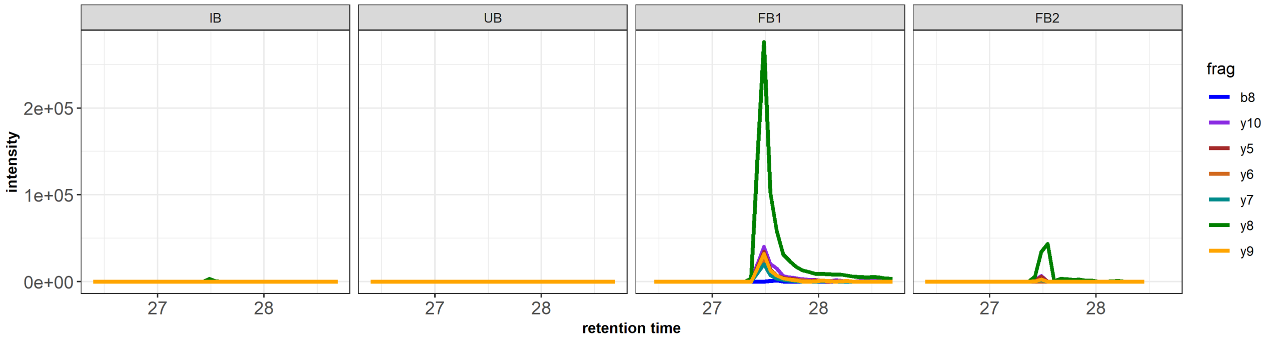

Supplement: Supplementary file 1 [file ijms-23-05061-s001.zip › a171-SupplFigure-S1.v220318.pdf]
