# Supplementary material for: Novel Antibody–Peptide Binding Assay Indicates Presence of Immunoglobulins against EGFR Phospho-Site S1166 in High-Grade Glioma
Source: Int J Mol Sci. 2022 May 2;23(9):5061. doi: 10.3390/ijms23095061 (PMC9100080; doi:10.3390/ijms23095061)

Supplemental Figure S2: LC-MS Peak plots of GFAP applicability experiment

Figure S2A: GFAP peptide RS[+80]YVSSGEMMVGGLAPGR

LC-MS chromatograms (PRM) of GFAP phospho-peptides RS[+80]YVSSGEMMVGGLAPGR measured with PRM in the IgG-bound (IB), unbound (UB), and filter bound (FB) fraction of the Ab-peptide binding assay. Colors in the chromatogram plots indicate the various fragments detected and used for quantification. Grey peak background indicates if a positive peak detection on basis of at least 3 fragments was achieved.

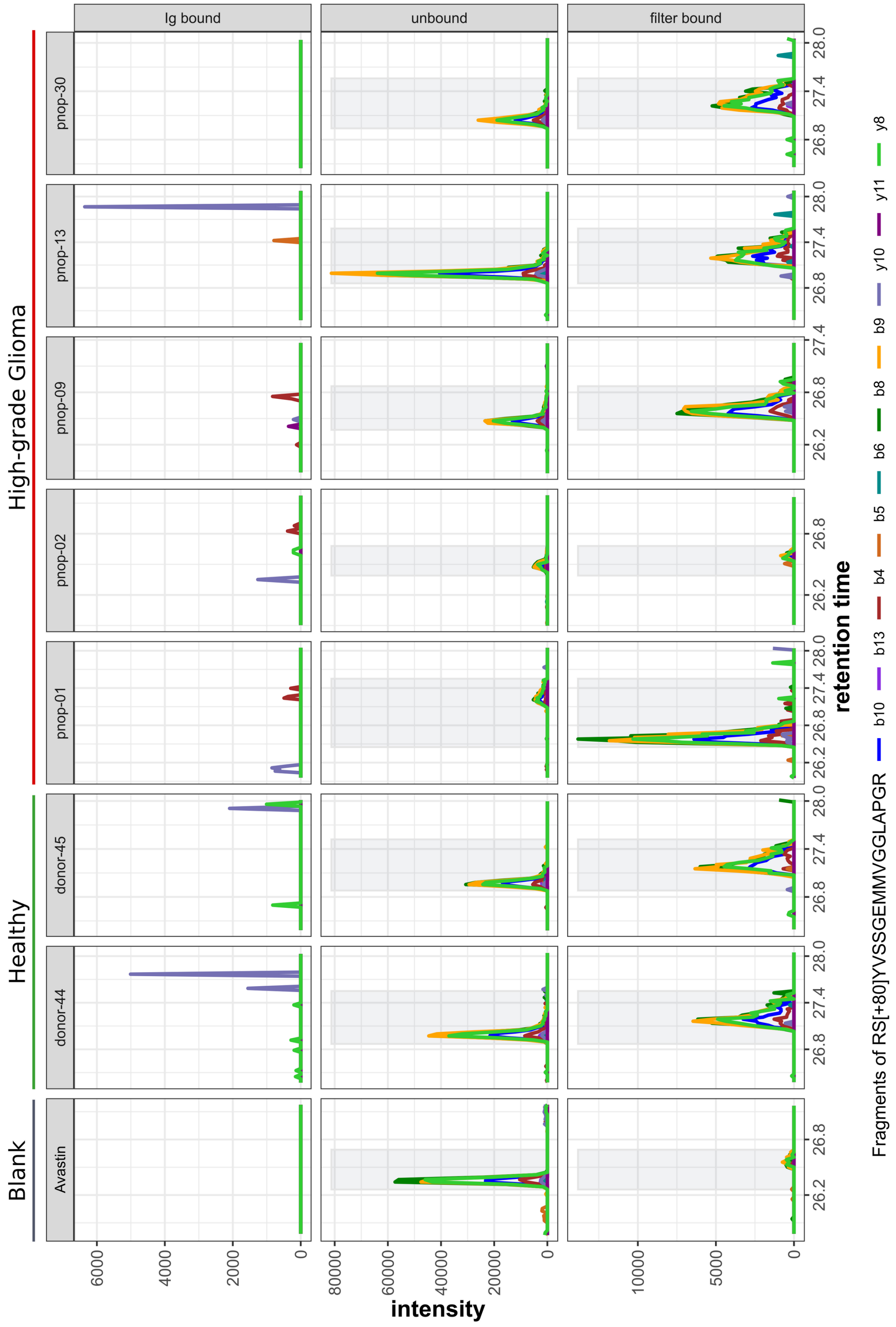

Figure S2B: GFAP peptide EAAS[+80]YQEALAR

LC-MS chromatograms (PRM) of GFAP phospho-peptides EAAS[+80]YQEALAR measured with PRM in the IgG-bound (IB), unbound (UB), and filter bound (FB) fraction of the Ab-peptide binding assay. Colors in the chromatogram plots indicate the various fragments detected and used for quantification. Grey peak background indicates if a positive peak detection on basis of at least 3 fragments was achieved.

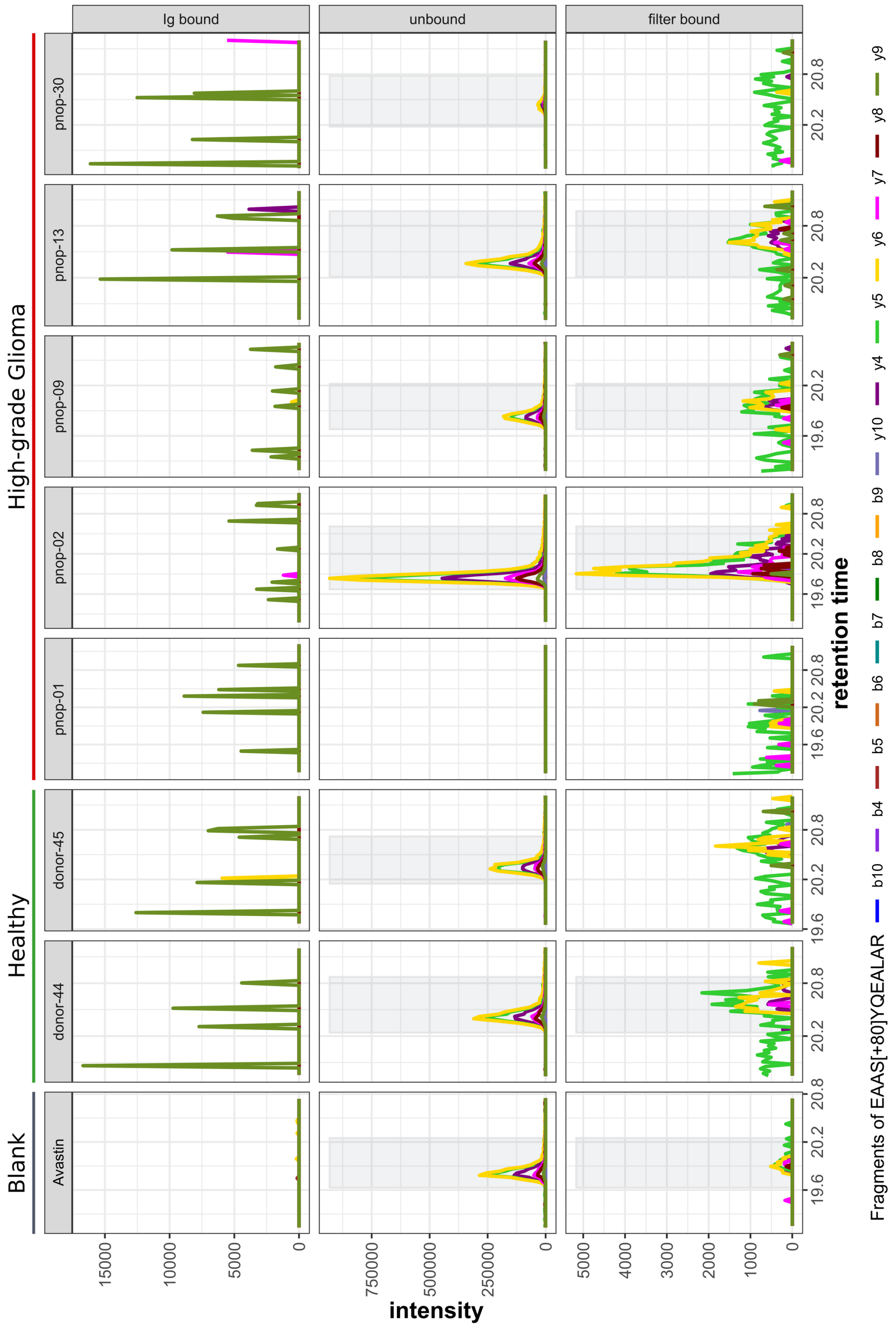

Figure S2C: GFAP peptide SVS[+80]EGHLK

LC-MS chromatograms (PRM) of GFAP phospho-peptides SVS[+80]EGHLK measured with PRM in the IgG-bound (IB), unbound (UB), and filter bound (FB) fraction of the Ab-peptide binding assay. Colors in the chromatogram plots indicate the various fragments detected and used for quantification. Grey peak background indicates if a positive peak detection on basis of at least 3 fragments was achieved.

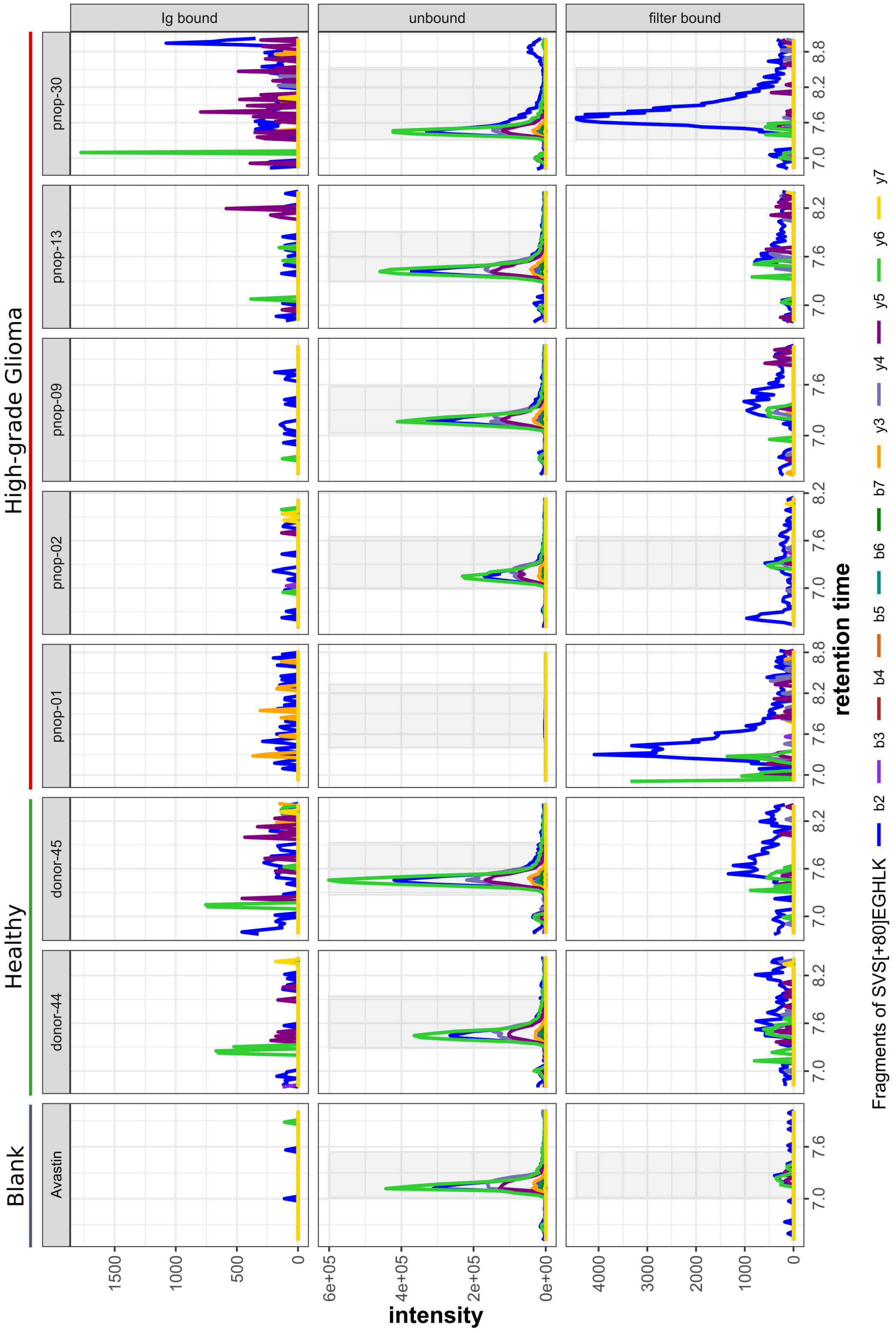

Supplement: Supplementary file 1 [file ijms-23-05061-s001.zip › a171-SupplFigure-S2.v220318B.pdf]
